# Supplementary material for: Complexome profiling of the Chlamydomonas psb28 mutant reveals TEF5 as an early PSII assembly factor
Source: Plant Cell. 2025 Mar 17;37(6):koaf055. doi: 10.1093/plcell/koaf055 (PMC12164590; doi:10.1093/plcell/koaf055)
Supplement: koaf055_Supplementary_Data [file koaf055_supplementary_data.zip › psb28_interactive.zip!/plots/intensity/qualityPlots/peptides/TPIC1_Cre01.g029300.t1.2.html]

### Cre01.g029300.t1.2

Trivial name: TPIC1  
SeqCoverage [median]: 4.30  
Mass: 30.05 kDa  
  
 

| Sequence | MissedCleavage | Mass [Da] | wtIntensity | muIntensity | Score |
| --- | --- | --- | --- | --- | --- |
| LVDELNAGTIPR | 0 | 1296.70 | 377.09 | 2464.41 | 211.68 |
|  |  |  | 377.1 | 2464.4 |  |
